# Efficacy of pulmonary surfactant with budesonide in infants born at or less than 28 weeks’ gestation: a systematic review and meta-analysis

**DOI:** 10.1038/s41598-025-33028-0

**Published:** 2025-12-22

**Authors:** Nanthida Phattraprayoon, Nut Koonrungsesomboon, Mingkwan Na Takuathung

**Affiliations:** 1 Princess Srisavangavadhana Faculty of Medicine, Chulabhorn Royal Academy, Bangkok, 10210 Thailand; 2 Department of Pharmacology, Faculty of Medicine, Chiang Mai University, Chiang Mai, 50200 Thailand; 3 Clinical Research Center for Food and Herbal Product Trials and Development (CR-FAH), Faculty of Medicine, Chiang Mai University, Chiang Mai, 50200 Thailand

**Keywords:** Budesonide, Bronchopulmonary dysplasia, Extremely preterm, Pulmonary surfactant, Medical research, Paediatric research

## Abstract

Pulmonary surfactant (PS) is the standard treatment for respiratory distress syndrome in preterm infants. Budesonide, a corticosteroid with anti-inflammatory properties, has been studied for use with PS to potentially improve respiratory outcomes and reduce bronchopulmonary dysplasia (BPD) risk while minimizing systemic steroid exposure. This study aimed to compare the efficacy of PS with budesonide versus PS alone in infants born ≤ 28 weeks’ gestational age (GA). A systematic review and meta-analysis was conducted by searching PubMed, Scopus, Embase, and the Cochrane Library databases from inception to October 21, 2025. Inclusion was restricted to randomized controlled trials (RCTs) examining PS with budesonide efficacy in extremely preterm populations. Risk ratios (RRs) or mean differences (MDs) with 95% confidence intervals (CIs) were calculated using random-effects models, and the quality of evidence was assessed with GRADE methodology. The primary outcomes were the incidence and severity of BPD; secondary outcomes included other respiratory measures, pre-discharge mortality, hospital stay duration, and complications related to prematurity. Compared to PS alone, PS with budesonide did not significantly reduce BPD incidence (RR, 0.96; 95% CI, 0.86 to 1.08; *p* = 0.51; I^2^ = 40%; low-certainty evidence), duration of mechanical ventilation (MD, − 0.45 days; 95% CI, − 2.26 to 1.36; *p* = 0.62; I^2^ = 0%; moderate-certainty evidence), pre-discharge mortality (RR, 0.92; 95% CI, 0.76 to 1.13; *p* = 0.44; I^2^ = 0%; low-certainty evidence), or duration of of hospitalization (MD, 0.27 days; 95% CI, − 3.29 to 3.84; *p* = 0.88; I^2^ = 0%; moderate-certainty evidence). PS with budesonide probably has little or no effect on BPD, respiratory outcomes, other prematurity-related outcomes, or pre-discharge mortality compared to surfactant alone. Most outcomes are based on low- to moderate-certainty evidence. Therefore, current evidence is insufficient to support the superior efficacy of pulmonary surfactant with budesonide over surfactant alone in extremely preterm infants.

**Supplementary Information:**

The online version contains supplementary material available at 10.1038/s41598-025-33028-0.

## Introduction

Extreme prematurity carries a substantial risk for respiratory complications due to pulmonary immaturity^1^. These complications, ranging from respiratory distress syndrome (RDS) to bronchopulmonary dysplasia (BPD), increase the risk of long-term pulmonary morbidity.The development of BPD is associated with multiple risk factors, including gestational age (GA), duration of mechanical ventilation, and hospital-acquired infections^2–5^.

The prevalence of BPD is substantially higher among extremely preterm infants^6^, ranging widely between 10% and 89% depending on geographic location^7^. Prevention of BPD remains crucial, utilizing evidence-based approaches such as antenatal corticosteroids, appropriate ventilatory management, pulmonary surfactant (PS) therapy with using optimized delivery techniques, postnatal steroid use, nutritional optimization, caffeine treatment, and vitamin A supplementation to improve neonatal outcomes^8–10^.

Despite advances in neonatal care that have improved survival rates, the incidence of BPD among extremely preterm infants remains high. Systemic corticosteroids are effective in reducing the risk of BPD; however, their use is limited by concerns regarding potential adverse neurodevelopmental outcomes, particularly when administered during the first week after birth^11^. For example, dexamethasone given during this early postnatal period has been associated with an increased risk of cerebral palsy and is not recommended^9^.

Consequently, PS with budesonide has been postulated as an alternative to improve outcomes and reduce complications in preterm infants. Accordingly, studies have investigated the use of PS with budesonide in preterm populations. The rationale for this approach is based on the established role of pulmonary inflammation in BPD pathogenesis^12,13^. Budesonide is a local anti-inflammatory corticosteroid that binds to glucocorticoid receptors in target cells, inhibiting inflammatory gene expression and reducing cytokine production, including interleukins^14^ and tumor necrosis factor (TNF)^15–17^. As budesonide is administered locally, it minimizes systemic exposure, thereby reducing the risk of adverse effects associated with systemic corticosteroid use, such as hyperglycemia and gastrointestinal bleeding.

Previous systematic reviews and meta-analyses^18,19^ have demonstrated that PS with budesonide reduces the incidence of BPD; however, these findings are primarily derived from studies involving very preterm infants. Considering the high burden of BPD in extremely preterm populations, this systematic review and meta-analysis aimed to determine whether PS with budesonide provides superior efficacy compared to PS alone specifically in infants with GA ≤ 28 weeks.

## Methods

### Data sources and searches

A comprehensive systematic search was conducted in PubMed, Scopus, Excerpta Medica database (Embase), and the Cochrane Library from their inception to October 21, 2025. The search terms included (“preterm” OR “premature”) AND [“surfactant” OR (“pulmonary surfactant”) OR (“exogenous surfactant”)] AND “budesonide.” References from the retrieved studies were reviewed to identify additional relevant studies.

This systematic review and meta-analysis was conducted in accordance with the Preferred Reporting Items for Systematic Reviews and Meta-Analyses (PRISMA) guidelines^20^, with the research protocol (CRD42024619815) registered in PROSPERO. An exemption certificate was obtained from the Research Ethics Committee of Princess Srisavangavadhana Faculty of Medicine, Chulabhorn Royal Academy (EC 015/2568).

### Eligibility criteria

This review included randomized controlled trials (RCTs) comparing PS with budesonide versus PS alone, without language restrictions. Inclusion criteria comprised studies that enrolled preterm infants with GA ≤ 28 weeks, and compared PS with budesonide (administered by any route) to PS alone, either for prophylaxis or treatment of RDS.

Two reviewers (NP and MN) independently assessed the literature for relevance, study design, methodology, and outcomes according to predetermined criteria. Discrepancies were resolved by a third researcher (NK) through discussion and consensus.

### Data extraction and quality assessment

The included studies reported the first author’s name, publication year, study design, participants’ country, intervention type, and outcomes. Our prespecified outcomes were as follows: The primary outcomes were the incidence and severity of BPD. Secondary outcomes included other respiratory measures, such as the requirement for postnatal systemic corticosteroids, pulmonary hemorrhage, and duration of mechanical ventilation. We also assessed other preterm-related complications, including pre-discharge mortality, length of hospital stay, late onset sepsis, patent ductus arteriosus (PDA) requiring treatment, retinopathy of prematurity (ROP), necrotizing enterocolitis (NEC), pneumothorax, and adverse drug reactions such as hyperglycemia, gastrointestinal bleeding, spontaneous intestinal perforation (SIP).

For missing or unclear data, the corresponding authors were contacted by email for clarification. The risk of bias was assessed using the revised Cochrane Risk of Bias tool for Randomized Trials (RoB 2)^21^, with studies categorized as having low risk, high risk, or some concerns. Bias assessments were visually summarized using the robvis tool.

### Data synthesis and statistical analysis

Random-effects models were employed to calculate risk ratios (RRs) for categorical outcomes and weighted mean differences (MDs) for continuous variables, with 95% confidence intervals (CIs). When studies reported data as median with interquartile range (IQR), established statistical conversion formulas were applied to transform these values into mean and standard deviation (SD) for analysis^22,23^.

Subgroup and sensitivity analyses were performed to investigate heterogeneity and evaluate the robustness of results. Between-study heterogeneity was assessed using the I² statistic, with values of 25%, 50%, and 75% considered to indicate low, moderate, and high heterogeneity across studies, respectively^24^. Publication bias was evaluated using funnel plots for analyses including ≥ 10 studies. A p-value < 0.05 was considered statistically significant, and all meta-analyses were conducted using RevMan software version 5.4.1.

The overall certainty of evidence for each outcome was assessed using the Grading of Recommendations Assessment, Development and Evaluation (GRADE) approach, which classifies quality of evidence as high, moderate, low, or very low^25,26^. This comprehensive assessment systematically evaluated five key domains: risk of bias^27^, inconsistency^28^, indirectness^29^, imprecision^30^, and publication bias^31^. The GRADE evidence profile tables and absolute effect estimates were generated using the GRADEpro Guideline Development Tool (http://gradepro.org)^32^.

### Definitions in this study

Definitions (where applicable) of the outcomes included in this systematic review and meta-analysis—including RDS, BPD, postnatal systemic corticosteroid requirement, pulmonary hemorrhage, pre-discharge mortality, late onset sepsis, PDA requiring treatment, SIP, and hyperglycemia—are provided in S1 Table.

## Results

### Search results

**Fig. 1:**
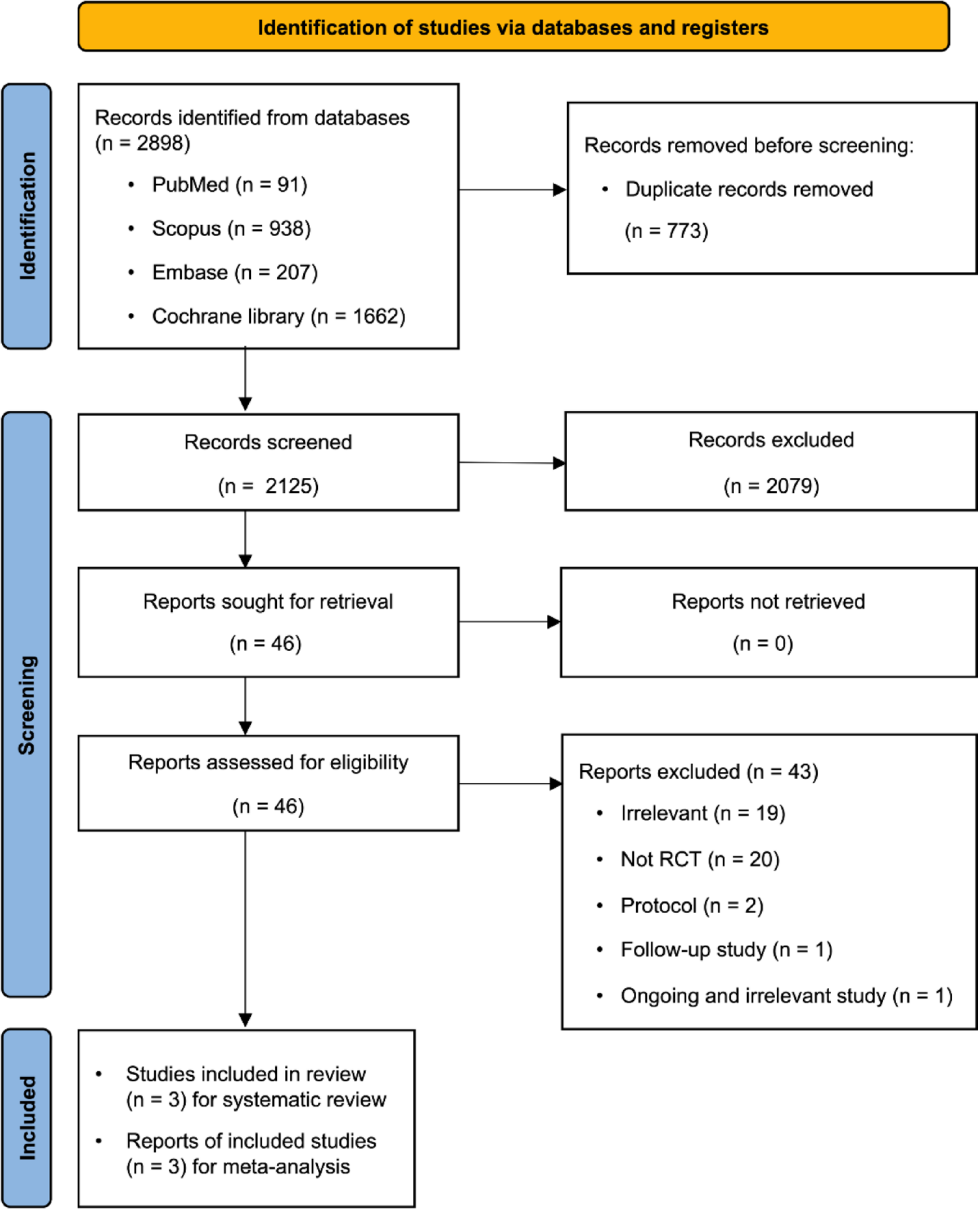
PRISMA flow diagram of the study selection for the systematic review and meta-analysis.

Our database search identified 2,898 records. After screening titles and abstracts, 46 full-text articles were assessed for eligibility. Among the 43 excluded studies, reasons for exclusion were irrelevance (*n* = 19), not RCTs (*n* = 20), study protocol (*n* = 2), follow-up study (*n* = 1), and study that was both ongoing and irrelevant (*n* = 1). Therefore, three studies^33–35^ were included in the systematic review and meta-analysis (Fig. 1, S5 Table).

### Study characteristics

Our meta-analysis included three RCTs with 1,768 participants: 880 infants receiving PS with budesonide and 888 receiving PS alone. Two studies administered PS with budesonide intratracheally (ITT) via an endotracheal tube (ETT)^34,35^ or catheter^34^, while one study delivered PS intratracheally through a catheter with nebulized budesonide^33^. Across these studies, PS was administered either via ETT^34,35^ or catheter for comparison^33,34^. The trials were conducted at a single center in Iran^33^, at 21 centers in Australia, New Zealand, Canada, and Singapore^34^, and at 17 centers in the United States of America^35^.

One study was published in 2018^33^ and two studies were published in the 2024–2025 period^34,35^. Rates of antenatal corticosteroid administration varied considerably: Sadeghnia et al. (2018), 38–48%; Manley et al., 95–96% (66–67% complete courses); and Ambalavanan et al., 99.1% (88.4–91.1% complete courses). Study characteristics and infant details are shown in Table 1 and S2 Table.

**Table 1:** The characteristics of the included studies.

Study	Type of study	Location	Inclusion criteria	Exclusion criteria	Randomization method	Study period	Intervention	*N* intervention	Control	*N* control
** Sadeghnia 2018** ^33^	RCT	Iran	- GA 23–28 weeks - RDS - Requiring CPAP with FiO_2_ > 0.4	- Congenital malformations - Prenatal asphyxia	Odd/even document numbers	Jun 2014– Apr 2016	PS 100 mg/kg ITT with budesonide 0.5 mg q 12 h NB (max. 7 days)	35	PS 100 mg/kg ITT	35
** Manley 2024** ^34^	RCT	Australia, New Zealand, Canada, and Singapore (multicenter study	- GA < 28 weeks - RDS required PS - Less than 48 h old - Requiring noninvasive respiratory support or mechanical ventilation	- Major congenital anomalies - Severe pulmonary hypoplasia - Received post-natal steroid before recruiting	Computer with permuted blocks	Jan 2018–Mar 2023 (last discharge in August 2023)	PS 200 mg/kg ITT with budesonide 0.25 mg/kg ITT 1–2 doses	524	PS 200 mg/kg ITT	535
** Ambalavanan 2025** ^35^	RCT	United states (multicenter study)	- GA 22–28 weeks - RDS required PS - BW 401–1000 g - ≤ 48 h old	Prior to enrollment: -Use of surfactant or systemic steroids - Known congenital infection - Serious chromosomal abnormality or malformation - Permanent neuromuscular disorder affecting respiration Indomethacin: -Maternal exposure within 24 h of delivery, or infant exposure before enrollment - Planned use within 7 days of last study drug dose Other: - Enrollment in a conflicting clinical trial	Step-forward randomization with a block urn design	Apr 2021- Jun 2024 (with early termination)	PS 200 mg/kg ITT with budesonide 0.25 mg/kg ITT 1–2 doses (additional open label PS dose up to 7 days)	323 Intention-to-treat for primary analysis (*n* = 321)	PS 200 mg/kg ITT	318 Intention-to-treat for primary analysis (*n* = 318)

Abbreviations: BW: birth weight; CPAP: continuous positive airway pressure; g: gram; FiO_2_: fraction of inspired oxygen; GA: gestational age; ITT: intratracheal; NB: nebulization; PS: pulmonary surfactant; RDS: respiratory distress syndrome; RCT: randomized controlled trial.

### Risk of bias assessment

Overall, in two studies^34,35^, allocation sequences were appropriately generated and concealed. All three studies implemented the intended interventions and adequately addressed participant loss to follow-up. Assessment of outcome measurement indicated either low risk^34,35^or some concerns^33^ across the studies, and result reporting was judged to be low risk in two studies^34,35^.

According to the revised Cochrane Risk of Bias (RoB 2) tool, two studies^34,35^ were rated as having low risk of bias across all outcomes, while one study was assessed as having some concerns for certain outcomes^33^. A detailed risk of bias assessment for each study and outcome is provided in S1–S11 Fig. 

### Primary outcomes

#### Incidence of BPD defined by the National Institutes of Health (NIH) consensus definition or Jensen et al. 2019 definition

**Fig. 2:**
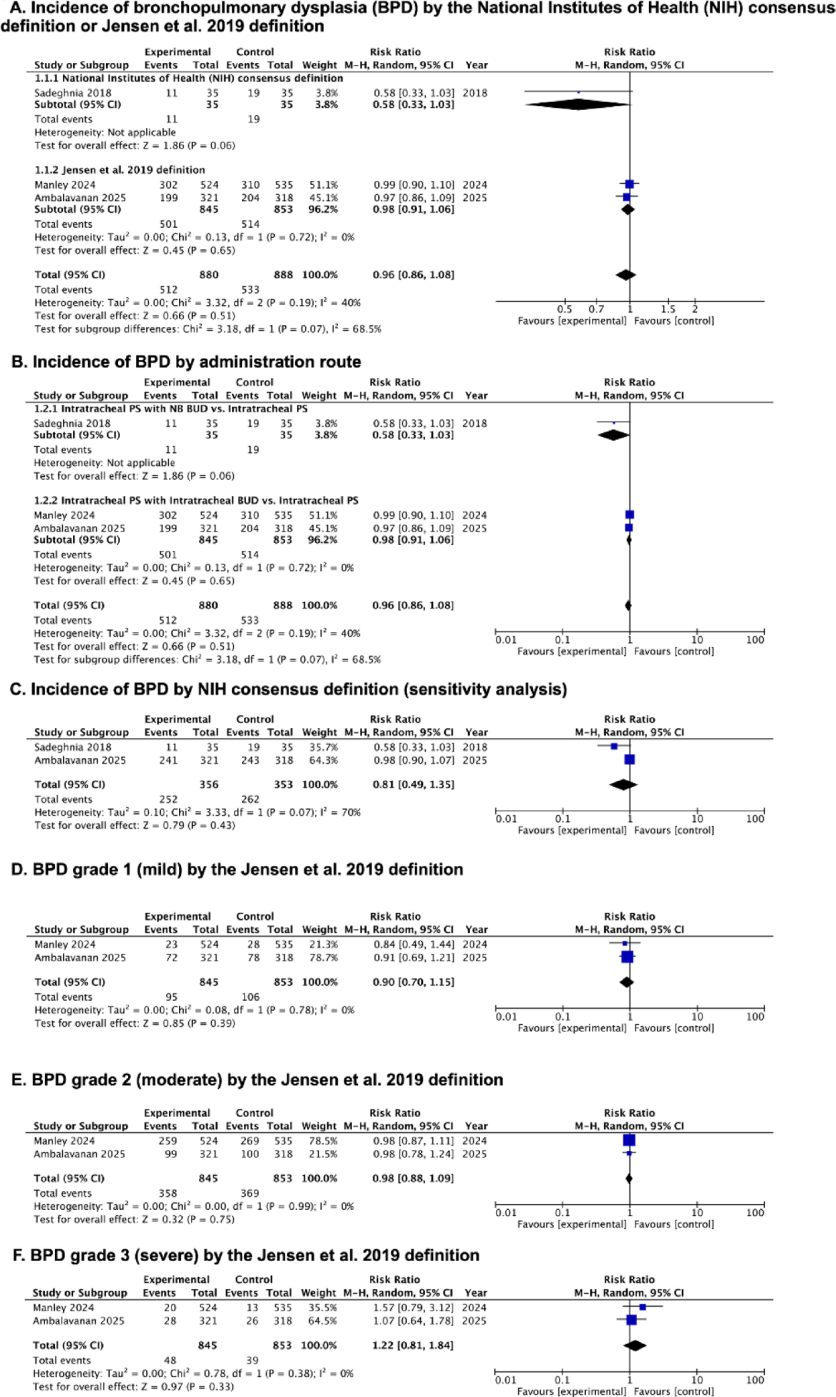
Forest plots of the efficacy of PS with budesonide in infants born at or less than 28 weeks’ gestation. **(A)** Incidence of bronchopulmonary dysplasia (BPD) defined by the National Institutes of Health (NIH) consensus definition or Jensen et al. 2019 definition; **(B)** Incidence of BPD by administration route; **(C)** Incidence of BPD defined by NIH consensus definition (sensitivity analysis); **(D)** BPD grade 1 (mild) defined by the Jensen et al. 2019 definition; **(E)** BPD grade 2 (moderate) defined by the Jensen et al. 2019 definition; **(F)** BPD grade 3 (severe) defined by the Jensen et al. 2019 definition.

Results from three RCTs were analyzed^33–35^. PS with budesonide did not significantly reduce the incidence of BPD compared to PS alone (RR, 0.96; 95% CI, 0.86 to 1.08; *p* = 0.51; I^2^ = 40%; 3 studies; 1768 participants; low-certainty evidence) (Fig. 2A; Table 2, S3 Table).

**Table 2:** GRADE summary of the findings regarding the efficacy of pulmonary surfactant with budesonide.

Patient or population: Extremely preterm Intervention: Pulmonary surfactant (PS) with budesonide Comparison: Pulmonary surfactant														
Study design	No. of studies	Certainty assessment							No. of participants			Effect		
		Risk of bias	Inconsistency		Indirectness	Imprecision		Other considerations	PS with Budesonide	PS		Estimation of absolute effects		Certainty
												**Relative** **(95% CI)**	**Absolute (95% CI)**	
**Primary outcomes**														
**Incidence of bronchopulmonary dysplasia defined by the National Institutes of Health (NIH) consensus definition or Jensen et al. 2019 definition**														
RCT	3	serious ^a^		not serious	not serious		serious ^d^	none	512/880 (58.2%)	533/888 (60.0%)	RR 0.96 (0.86 to 1.08)		24 fewer per 1,000 (from 84 fewer to 48 more)	⨁⨁◯◯ Low
**Severity of bronchopulmonary dysplasia defined by Jensen et al. 2019 definition**														
**Grade 1 (mild)**														
RCT	2	not serious		not serious	not serious		serious ^d^	none	95/845 (11.2%)	106/853 (12.4%)	RR 0.90 (0.70 to 1.15)		12 fewer per 1,000 (from 37 fewer to 19 more)	⨁⨁⨁◯ Moderate
**Grade 2 (moderate)**														
RCT	2	not serious		not serious	not serious		serious ^d^	none	358/845 (42.4%)	369/853 (43.3%)	RR 0.98 (0.88 to 1.09)		9 fewer per 1,000 (from 52 fewer to 39 more)	⨁⨁⨁◯ Moderate
**Grade 3 (severe)**														
RCT	2	not serious		not serious	not serious		serious ^d^	none	48/845 (5.7%)	39/853 (4.6%)	RR 1.22 (0.81 to 1.84)		10 more per 1,000 (from 9 fewer to 38 more)	⨁⨁⨁◯ Moderate
**Secondary outcomes**														
**Other respiratory outcomes**														
**Postnatal systemic corticosteroid requirement**														
RCT	2	not serious		not serious	not serious		serious ^d^	none	276/845 (32.7%)	285/853 (33.4%)	RR 0.98 (0.85 to 1.12)		7 fewer per 1,000 (from 50 fewer to 40 more)	⨁⨁⨁◯ Moderate
**Pulmonary hemorrhage**														
RCT	2	not serious		not serious	not serious		serious ^d^	none	49/846 (5.8%)	70/848 (8.3%)	RR 0.71 (0.50 to 1.00)		24 fewer per 1,000 (from 41 fewer to 0 fewer)	⨁⨁⨁◯ Moderate
**Duration of mechanical ventilation (days)**														
RCT	2	not serious		not serious	not serious		serious ^d^	none	839	839	-		MD 0.45 lower (2.26 lower to 1.36 higher)	⨁⨁⨁◯ Moderate
**Other preterm outcomes**														
**Pre-discharge mortality**														
RCT	3	serious^a^		not serious	not serious		serious ^d^	none	151/880 (17.2%)	164/883 (18.6%)	RR 0.92 (0.76 to 1.13)		15 fewer per 1,000 (from 45 fewer to 24 more)	⨁⨁◯◯ Low
**Duration of hospitalization (days)**														
RCT	2	not serious		not serious	not serious		serious ^d^	none	737	724	-		MD 0.27 higher (3.29 lower to 3.84 higher)	⨁⨁⨁◯ Moderate
**Late onset sepsis**														
RCT	2	not serious		not serious	not serious		serious ^d^	none	145/846 (17.1%)	150/848 (17.7%)	RR 0.96 (0.78 to 1.19)		7 fewer per 1,000 (from 39 fewer to 34 more)	⨁⨁⨁◯ Moderate
**Patent ductus arteriosus requiring treatment**														
RCT	2	not serious		not serious	serious^c^		serious ^d^	none	223/843 (26.5%)	254/843 (30.1%)	RR 0.88 (0.75 to 1.02)		36 fewer per 1,000 (from 75 fewer to 6 more)	⨁⨁◯◯ Low
**Adverse effects**														
**Hyperglycemia**														
RCT	2	not serious		serious^b^	not serious		serious ^d^	none	554/846 (65.5%)	487/848 (57.4%)	RR 1.18 (0.93 to 1.49)		103 more per 1,000 (from 40 fewer to 281 more)	⨁⨁◯◯ Low
**Spontaneous Intestinal Perforation (SIP)**														
RCT	2	not serious		not serious	not serious		serious ^d^	none	36/846 (4.3%)	26/848 (3.1%)	RR 1.38 (0.84 to 2.27)		12 more per 1,000 (from 5 fewer to 39 more)	⨁⨁⨁◯ Moderate

^a^ Downgraded by one level for risk of bias due to the randomization process and/or outcome assessment, including possible selection of the reported result.

^b^ Downgraded by one level for inconsistency due to substantial heterogeneity (I² = 50%).

^c^ Downgraded by one level due to indirect effects; potential influence from other factors.

^d^ Downgraded by one level for imprecision as the 95% confidence interval includes both potential benefit and harm.

Abbreviations: CI: confidence interval; PS: pulmonary surfactant; RCTs: randomized controlled trials; RR: risk ratio.

GRADE certainty of the evidence.

High: We are very confident that the true effect lies close to that of the estimate of the effect.

Moderate: We are moderately confident in the effect estimate: the true effect is likely to be close to the estimate of the effect, but there is a possibility that it is substantially different.

Low: Our confidence in the effect estimate is limited; the true effect may be substantially different from the estimate of the effect.

Very low: We have very little confidence in the effect estimate; the true effect is likely to be substantially different from the estimate of the effect.

In a subgroup analysis based on the type of budesonide intervention and the definition of BPD—either the National Institutes of Health (NIH) consensus definition or the Jensen et al. (2019) definition. Based on the type of budesonide intervention, two studies administered PS with budesonide intratracheally via ETT^34,35^ or catheter^34^, while one study^33^ delivered intratracheal PS via catheter with nebulized budesonide. The pooled estimate for PS with budesonide delivered intratracheally did not demonstrate a significant effect on the incidence of BPD (RR, 0.98; 95% CI, 0.91 to 1.06; *p* = 0.65; I^2^ = 0%; 2 studies; 1698 participants) (Fig. 2B).

Sensitivity analyses for BPD outcomes were performed using NIH consensus definition as applied in Sadeghnia et al. and Ambalavanan et al.; however, these analyses did not show a significant effect on the incidence of BPD (RR, 0.81; 95% CI, 0.49 to 1.35; *p* = 0.43; I^2^ = 70%; 2 studies; 709 participants) (Fig. 2C).

#### BPD grading defined by Jensen et al. 2019 definition

Two studies^34,35^ reported the incidence of severity of BPD using Jensen et al. 2019 definition (Fig. 2D, E and F; Table 2, S3 Table).

#### Grade 1 (mild)

The pooled meta-analysis of grade 1 BPD showed no significant difference in incidence between the intervention and control groups (RR, 0.90; 95% CI, 0.70 to 1.15; *p* = 0.39; I² = 0%; 2 studies; 1698 participants; moderate-certainty evidence).

#### Grade 2 (moderate)

The pooled meta-analysis of grade 2 BPD demonstrated no significant difference in incidence between the intervention and control groups (RR, 0.98; 95% CI, 0.88 to 1.09; *p* = 0.75; I² = 0%; 2 studies; 1698 participants; moderate-certainty evidence).

#### Grade 3 (severe)

The pooled results of the meta-analysis for grade 3 BPD showed no statistically significant difference in the incidence rates between the intervention and control groups (RR, 1.22; 95% CI, 0.81 to 1.84; *p* = 0.33; I² = 0%; 2 studies; 1698 participants; moderate-certainty evidence).

### Secondary outcomes

#### Other respiratory outcomes

##### Postnatal systemic corticosteroid requirement

**Fig. 3:**
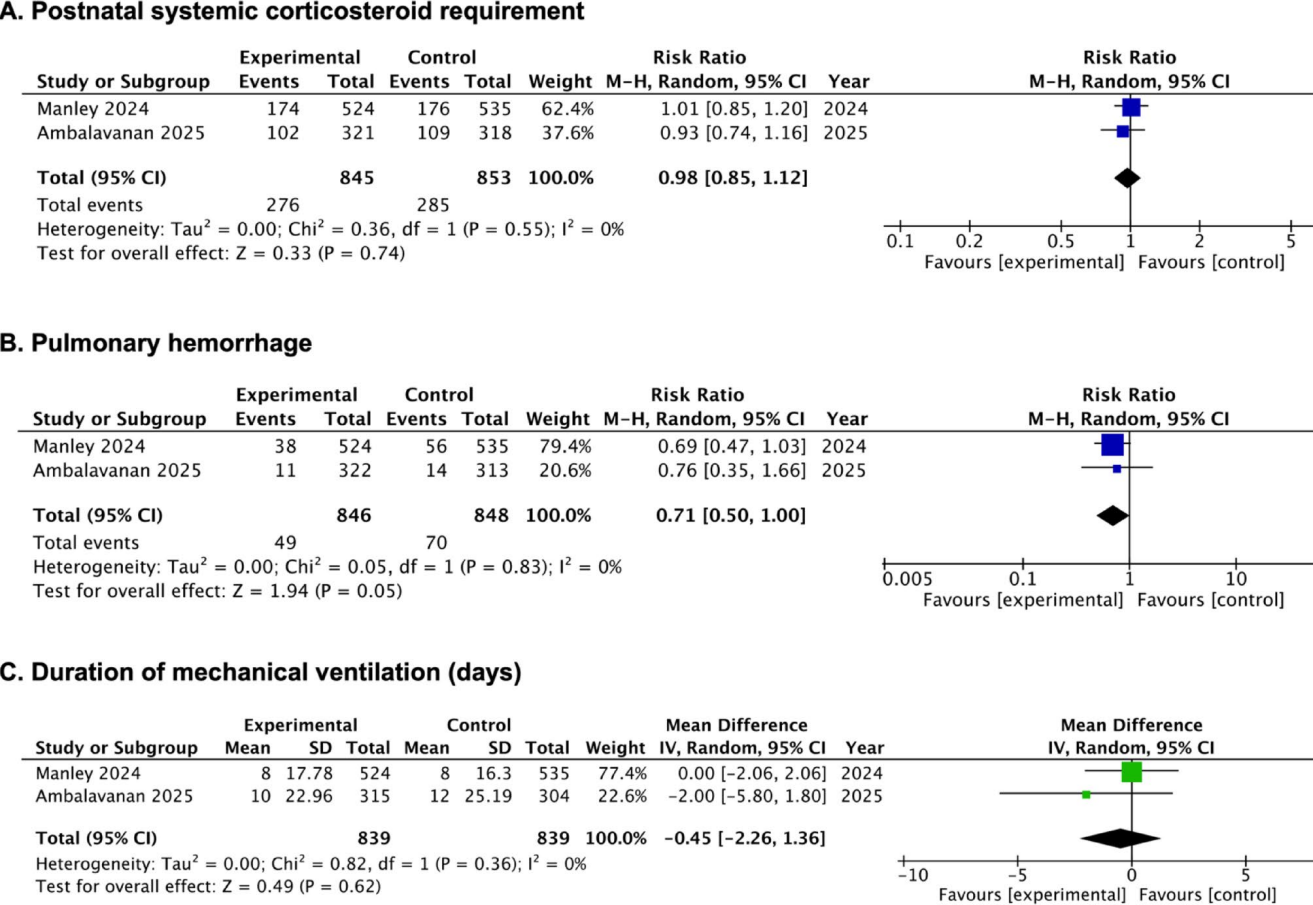
Forest plots of the efficacy of PS with budesonide in infants born at or less than 28 weeks’ gestation. **(A)** Postnatal systemic corticosteroid requirement; **(B)** Pulmonary hemorrhage; **(C)** Duration of mechanical ventilation (days).

No significant difference in the requirement for postnatal systemic steroids was observed between the intervention and control groups based on two RCTs^34,35^(RR, 0.98; 95% CI, 0.85 to 1.12; *p* = 0.74; I^2^ = 0%; 2 studies; 1698 participants; moderate-certainty evidence) in two RCTs^34,35^(Fig. 3A; Table 2, S3 Table).

##### Pulmonary hemorrhage

There was no statistically significant difference in the incidence of pulmonary hemorrhage between infants who received PS with budesonide and those in the control group (RR, 0.71; 95% CI, 0.50 to 1.00; *p* = 0.05; I^2^ = 0%; 2 studies; 1694 participants; moderate-certainty evidence) based on two studies^34,35^ (Fig. 3B; Table 2, S3 Table).

##### Duration of mechanical ventilation (MV) (days)

Two RCTs^34,35^ reported on duration of MV. No statistically significant reduction in MV duration was observed with PS plus budesonide compared to the control group (MD, −0.45 days; 95% CI, −2.26 to 1.36 days; *p* = 0.62; I^2^ = 0%; 2 studies; 1678 participants; moderate-certainty evidence) (Fig. 3C; Table 2, S4Table). Both Manley et al^34^. and Ambalavanan et al.^35^ reported this result as median and IQR. The data were converted to mean and SD using the statistical methods described in the methodology section.

#### Other preterm outcomes

##### Pre-discharge mortality

**Fig. 4:**
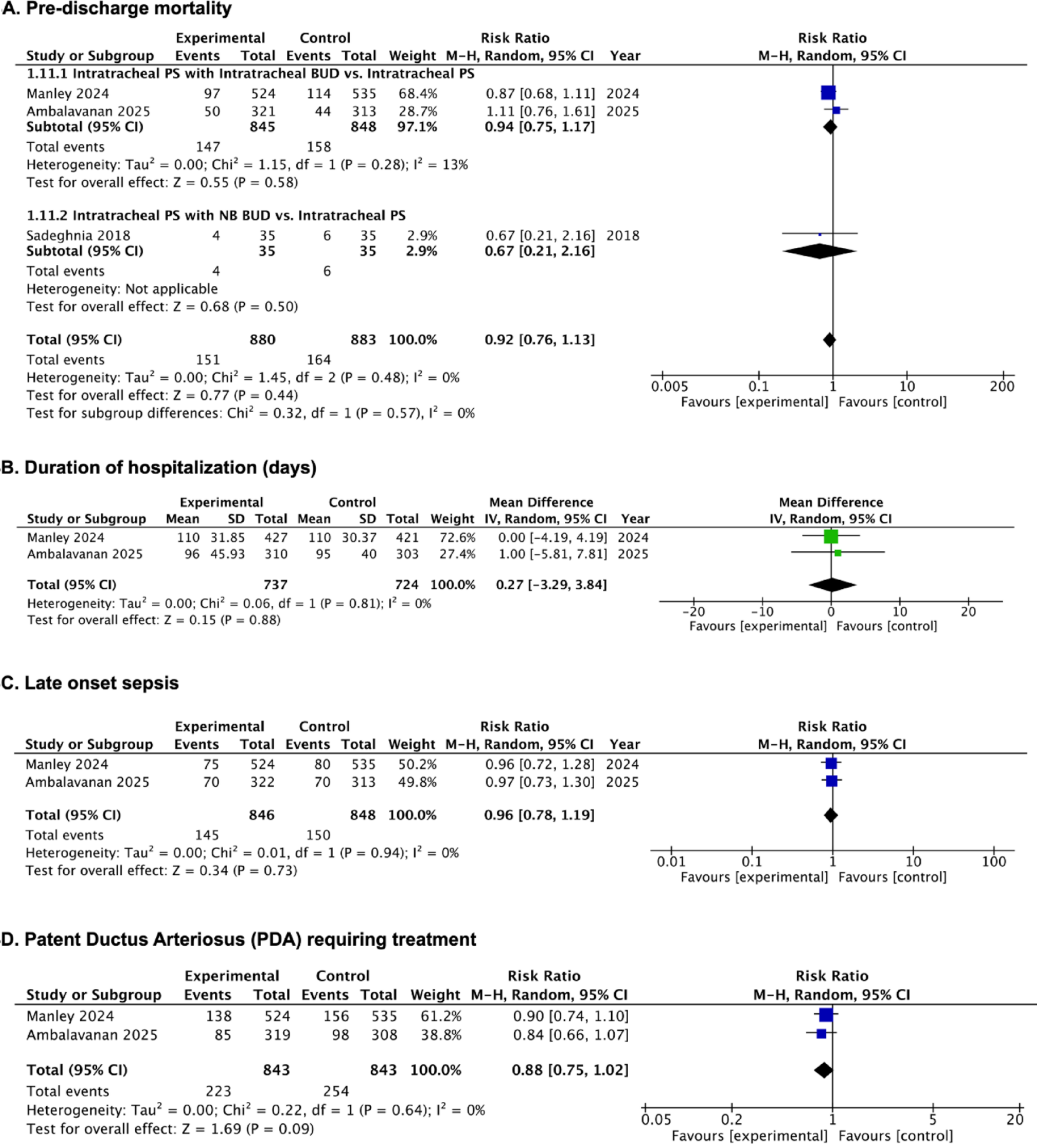
Forest plots of the efficacy of PS with budesonide in infants born at or less than 28 weeks’ gestation. **(A)** Pre-discharge mortality; **(B)** Duration of hospitalization (days); **(C)** Late onset sepsis; **(D)** Patent Ductus Arteriosus (PDA) requiring treatment.

Pre-discharge mortality did not differ significantly between the groups receiving PS with budesonide and the control group, based on three RCTs^33–35^ (RR, 0.92; 95% CI, 0.76 to 1.13; *p* = 0.44; I^2^ = 0%; 3 studies; 1763 participants; low-certainty evidence) (Fig. 4A; Table 2, S3 Table).

##### Duration of hospitalization (days)

No significant reduction in hospital stay duration was observed in the group of patients treated with PS and budesonide compared to the control group (MD, 0.27 days; 95% CI, − 3.29 to 3.84 days; *p* = 0.88; I^2^ = 0%; 2 studies; 1461 participants; moderate-certainty evidence)^34,35^ (Fig. 4B; Table 2, S4 Table). However, both Manley et al.^34^ and Ambalavanan et al.^35^ originally reported their results as median and IQR.

##### Late onset sepsis

The incidence of late onset sepsis was not significantly different between the group receiving PS with budesonide and the control group, based on a pooled analysis of two RCTs^34,35^ (RR, 0.96; 95% CI, 0.78 to 1.19; *p* = 0.73; I^2^ = 0%; 2 studies; 1694 participants; moderate-certainty evidence) (Fig. 4C; Table 2, S3 Table).

##### PDA requiring treatment

There was no significant difference in this outcome between the groups receiving PS with budesonide and the control group, based on two RCTs^34,35^ (RR, 0.88; 95% CI, 0.75 to 1.02; *p* = 0.09; I^2^ = 0%; 2 studies; 1686 participants; low-certainty evidence) (Fig. 4D; Table 2, S3 Table).

### Adverse effects

**Fig. 5:**
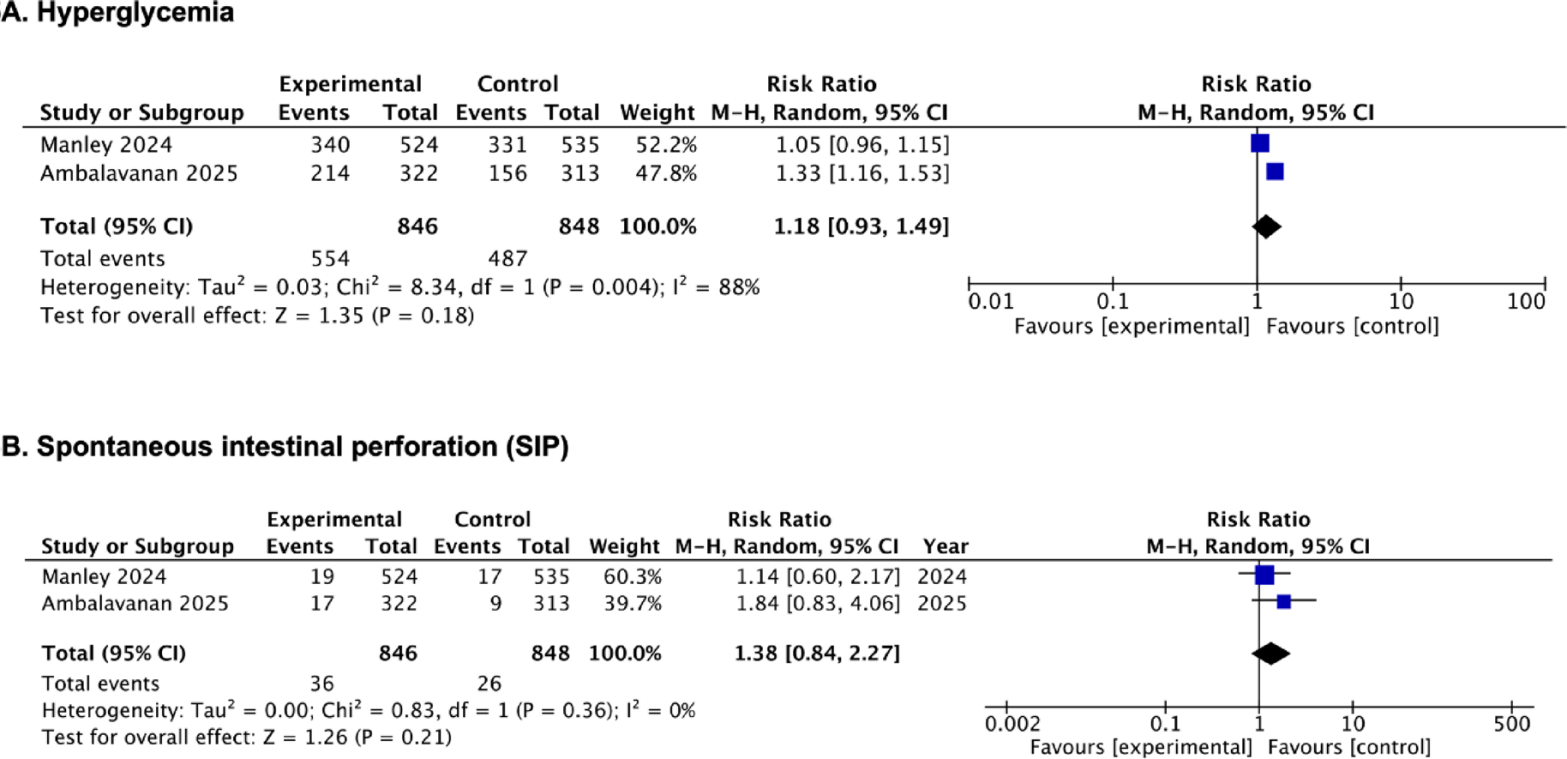
Forest plots of the efficacy of PS with budesonide in infants born at or less than 28 weeks’ gestation. **(A)** Hyperglycemia; **(B)** Spontaneous intestinal perforation (SIP).

Adverse effects were reported by a two studies^34,35^. Pooled estimates showed no significant between-group differences for hyperglycemia (RR, 1.18; 95% CI, 0.93 to 1.49; *p* = 0.18; I^2^ = 88%; 2 studies; 1694 participants; low-certainty evidence) and spontaneous intestinal perforation (RR, 1.38; 95% CI, 0.84 to 2.27; *p* = 0.21; I^2^ = 0%; 2 studies; 1694 participants; moderate-certainty evidence) (Fig. 5A and B; Table 2, S3 Table).

### Other pre-specified outcomes

Other prespecified outcomes—included repeated doses of PS, ROP, NEC, pneumothorax, and gastrointestinal bleeding—were intended for pooled analysis; however, due to inconsistent reporting among the included studies, meta-analysis for these outcomes could not be performed.

## Discussion

Our systematic review and meta-analysis specifically included studies enrolling infants with a gestational age of ≤ 28 weeks. Overall, we found no significant reduction in the incidence or severity of BPD when PS was administered with budesonide compared to PS alone, in both overall and subgroup analyses. Subgroup analyses based on the route of budesonide administration also did not show a significant reduction in BPD incidence compared to PS alone.

The evolving definition of BPD presents challenges for comparing findings across studies. The traditional NIH consensus definition classifies BPD based on the requirement for respiratory support for at least 28 days and the level of respiratory support at 36 weeks’ postmenstrual age (PMA)^36^, whereas the newer Jensen et al. definition—now increasingly adopted—defines BPD as the need for respiratory support at 36 weeks’ PMA, with no BPD if no support is required^37^. This change may result in different rates of BPD diagnosis. Among the included studies, Sadeghnia et al.^33^ applied the NIH consensus definition, Manley et al. utilized the Jensen et al. 2019 definition, and Ambalavanan et al. used both NIH consensus definition and the Jensen et al. 2019 definition; accordingly, subgroups also were created based on the BPD definition used. In a sensitivity analysis (Ambalavanan et al. and Sadeghnia et al.) for the NIH consensus definition, no significant difference in BPD outcome was observed.

Severity grading of BPD using the Jensen et al. 2019 definition did not show significant differences across all grades (mild, moderate, severe) between infants receiving PS with budesonide and those receiving PS alone. Besides BPD outcomes, other respiratory outcomes—including postnatal systemic corticosteroid use, pulmonary hemorrhage, pre-discharge mortality, late onset sepsis, PDA requiring treatment, duration of mechanical ventilation, and length of hospitalization—were also not significantly different between the two groups of extremely preterm infants. Adverse events, such as hyperglycemia and SIP, did not differ in the pooled meta-analysis.

A key distinction in our meta-analysis is the focus on infants ≤ 28 weeks’ gestation, in contrast to previous studies, which included more mature preterm infants (gestational age > 28 weeks; birth weight > 1,000 g). Because GA strongly influences prognosis and BPD risk^38^, week-to-week differences can have a significant impact on outcomes. Fetal lung development progresses continuously throughout gestation^39^. Between 17 and 26 weeks’ gestational age, the lungs are in the canalicular stage. At 27 weeks, infants transition to the saccular stage, but the lungs remain immature and require intensive respiratory support for adequate function^5,40^.

In previous meta-analysis by Phattraprayoon et al.^18^, benefits of budesonide with PS were reported. The majority of infants in the included studies had gestational ages above 28 weeks. Although some studies, such as those by Yeh et al. (2008, 2016)^41,42^, enrolled infants both below and above 28 weeks’ gestation, approximately 27–38% of participants in Yeh et al. (2016)^42^ had birth weights exceeding 1,000 g. Therefore, the findings in previous meta-analysis may primarily reflect outcomes in preterm rather than extremely preterm infants. Additionally, both Yeh et al. studies^41,42^ were conducted over a decade ago, during a period of significant advances in neonatal care—particularly in ventilator management and BPD prevention strategies—compared to studies by Manley et al.^34^ and Ambalavanan et al.^35^, which were conducted in the current era. Furthermore, extremely preterm infants encounter additional inflammatory and multifactorial challenges—including delivery room intubation, increased requirements for invasive ventilation over a prolonged period, extended hospitalization, and postnatal growth failure—all of which contribute to BPD development^43^. These risk factors may be less prevalent in more mature preterm infants.

A strength of this study is its focused evaluation of extremely preterm infants, representing the highest-risk population. Nonetheless, several limitations should be noted: first, substantial variability exists in BPD definitions (NIH consensus definition vs. Jensen et al. 2019 definition); second, differences in the route of budesonide administration (intratracheal vs. nebulized) may impact comparability; when budesonide is mixed with PS and administered intratracheally, the surfactant serves as a delivery vehicle, facilitating transport of the drug to peripheral lung regions via the Marangoni effect and thereby enhancing both pulmonary deposition and anti-inflammatory activity^38,44^; and third, only three studies met the inclusion criteria—including one with a small sample size—while the majority of data were contributed by the trials of Manley et al. (2024)^34^ and Ambalavanan et al. (2025)^35^.

Further large-scale, well-designed randomized controlled trials are needed to more definitively evaluate the efficacy of PS with budesonide compared with PS alone in extremely preterm infants. Future research using unified definitions of BPD and related outcomes, standardized administration protocols, and consistent outcome reporting would help strengthen the reliability and interpretability of pooled meta-analyses and better clarify the benefits of this intervention in extremely preterm infants. Additionally, incorporating long-term follow-up assessments—including neurodevelopmental outcomes—would provide a more comprehensive understanding of the intervention’s overall impact.

## Conclusions

Our findings indicate that for infants born at ≤ 28 weeks’ gestation, current evidence does not yet support the routine use of PS with budesonide over surfactant alone. Further well-designed randomized controlled trials are needed to clarify the efficacy and safety of pulmonary surfactant with budesonide in extremely preterm infants.

## Supplementary Information

Below is the link to the electronic supplementary material.

Supplementary Material 1

Supplementary Material 2

## Data Availability

All data relevant to the study are included in the article or have been uploaded as supplementary information.
