# Supplementary material for: Efficacy of pulmonary surfactant with budesonide in infants born at or less than 28 weeks’ gestation: a systematic review and meta-analysis
Source: Sci Rep. 2025 Dec 22;15:45116. doi: 10.1038/s41598-025-33028-0 (PMC12748727; doi:10.1038/s41598-025-33028-0)
Supplement: Supplementary file 1 — Supplementary Material 1 [file 41598_2025_33028_MOESM1_ESM.pdf]

S1 Table. Definitions in this study

**Respiratory Distress Syndrome (RDS)**<br>RDS was diagnosed based on clinical and radiographic features
**Bronchopulmonary Dysplasia (BPD) and Chronic Lung Disease (CLD)**<br>These included studies that used interchangeable terms for Bronchopulmonary Dysplasia (BPD) and Chronic Lung Disease (CLD). Sadeghnia et al. referred to CLD, Manley et al. and Ambalavnan et al. used the term BPD.<br>BPD or CLD is defined as a condition in infants who experience respiratory distress at birth and require oxygen (>21%) at >28 days of age [1]. One study used new BPD definition (Jensen et al. 2019 definition) and defined “no BPD” as an infant who required no respiratory support or supplemental oxygen at discharge prior to 36 weeks postmenstrual age (PMA) [2]. While Ambalavanan et al.[3] used both National Institutes of Health (NIH) consensus definition and Jensen et al. 2019 definition for BPD.<br>The grading of BPD severity used in Manley et al. and Ambalavanan et al. followed the new BPD classification proposed by Jensen et al. 2019 definition.
**Postnatal systemic corticosteroid requirement**<br>Postnatal systemic corticosteroid requirement was defined as participants who received dexamethasone or corticosteroids after birth.
**Pulmonary hemorrhage**<br>In the study by Manley et al.,[2] pulmonary hemorrhage was defined using a cutoff of less than 48 hours after the first intervention, whereas Ambalavanan et al.[3] used a cutoff of 7 days.
**Pre-discharge mortality**<br>Mortality was defined as participants who died at any time during hospitalization.
**Late onset sepsis**

Manley et al. [2] defined sepsis as occurring after 48 hours of age, with a positive bacterial or fungal culture from blood or cerebrospinal fluid, or a negative blood culture but clinical suspicion of sepsis, with treatment using antibiotics or antifungals for at least 5 days. Manley et al. [2] used a cutoff for late-onset sepsis as an adverse effect, defined as occurring within 14 days following the intervention, whereas Ambalavanan et al. [3] used a cutoff of 7 days after the last dose of the study drug.
**Patent Ductus Arteriosus (PDA) requiring treatment**<br>Defined as a patient with a patent ductus arteriosus requiring treatment with medication and/or surgical ligation [2,3].
**Spontaneous Intestinal Perforation (SIP)**<br>Ambalavanan et al.[3] defined the cutoff as 30 days after the last dose of the study drug.
**Hyperglycemia**<br>Manley et al. [2] used a cutoff of >180 mg/dL and/or receiving insulin therapy within 14 days after the first intervention, whereas Ambalavanan et al. [3]monitored these events for 7 days after the last dose of the study drug.

References

1 Sadeghnia, A., Beheshti, B. K. & Mohammadizadeh, M. The Effect of Inhaled Budesonide on the Prevention of Chronic Lung Disease in Premature Neonates with Respiratory Distress Syndrome. Int J Prev Med 9, 15, doi:10.4103/ijpvm.IJPVM_336_16 (2018).

2 Manley, B. J. et al. Intratracheal Budesonide Mixed With Surfactant for Extremely Preterm Infants: The PLUSS Randomized Clinical Trial. Jama, doi:10.1001/jama.2024.17380 (2024).

3 Ambalavanan, N. et al. Early Intratracheal Budesonide to Reduce Bronchopulmonary Dysplasia in Extremely Preterm Infants: The Budesonide in Babies (BiB) Randomized Clinical Trial. JAMA, doi:10.1001/jama.2025.16450 (2025).

S2 Table. Baseline maternal and neonatal characteristics of the participants in the included studies

Characteristics	Sadeghnia 2018		Manley 2024		Ambalavanan 2025	
	**Intervention**	**Control**	**Intervention**	**Control**	**Intervention**	**Control**
**Number of participants**	35	35	524	535	323	318
**Maternal age (years) mean ± SD**	NR	NR	31.1 ± 6.3	30.9 ± 5.9	29.1 ± 6.1	29.7 ± 5.8
**Cesarean delivery, n (%)**	26 (76.5)	25 (71.4)	328 (62.5)	353 (65.9)	243 (75.7)**	236 (74.2)
**Prenatal corticosteroids, n (%)**	13 (38.2)	17 (48.6)	501 (95.6)	515 (96.3)	318 (99.1)**	314 (99.1)***
**GA (weeks), mean ± SD**	26.9 ± 1.3	27.3 ± 0.8	25.7 ± 1.3	25.6 ± 1.4	25.8 ± 1.9	25.9 ± 2.0
**Birth weight (g), mean ± SD**	902.9 ± 117.6	928.4 ± 143.7	768 ± 197.04*	740 ± 211.85*	808.1 ± 268.8	812.3 ± 242.6
**Male, n(%)**	17 (48.6)	15 (42.9)	288 (55)	298 (55.7)	158 (49.2)**	162 (50.9)
**Small for gestational age, n (%)**	NR	NR	73 (13.9)	74 (13.8)	62 (19.3)**	50 (15.7)

Abbreviations: g: gram; GA: Gestational age; NR: No report; SD: standard deviation
* Converted data from median (IQR 1-3) to mean (SD)
** Data were based on 321 participants.
*** Data were based on 317 participants.
Data extractors: NP and MN; The date of data extraction: October 25-30, 2025

S3 Table. Summary results of the included studies categorized by outcomes (incidence)

Author (year)	Intervention	PS with budesonide		PS (control)		Risk Ratio (95% CI)
		Event	Total	Event	Total	
**Primary outcomes**						
**Incidence of Bronchopulmonary Dysplasia defined by the National Institutes of Health (NIH) consensus definition or Jensen et al. 2019 definition**						
Sadeghnia 2018	PS ITT with budesonide NB	11	35	19	35	0.58 [0.33, 1.03]
Manley 2024	PS ITT with budesonide ITT	302	524	310	535	0.99 [0.90, 1.10]
Ambalavanan 2025	PS ITT with budesonide ITT	199	321	204	318	0.97 [0.86, 1.09]
Total (95% CI)		512	880	533	888	0.96 [0.86, 1.08]
Heterogeneity: Tau² = 0.00; Chi² = 3.32, df = 2 (P = 0.19); I² = 40%<br>Test for overall effect: Z = 0.66 (P = 0.51)						
**Severity of bronchopulmonary dysplasia defined by Jensen et al. 2019 definition**						
**Grade 1 (mild)**						
Manley 2024	PS ITT with budesonide ITT	23	524	28	535	0.84 [0.49, 1.44]
Ambalavanan 2025	PS ITT with budesonide ITT	72	321	78	318	0.91 [0.69, 1.21]
Total (95% CI)		95	845	106	853	0.90 [0.70, 1.15]
Heterogeneity: Tau² = 0.00; Chi² = 0.08, df = 1 (P = 0.78); I² = 0%<br>Test for overall effect: Z = 0.85 (P = 0.39)						
**Grade 2 (moderate)**						
Manley 2024	PS ITT with budesonide ITT	259	524	269	535	0.98 [0.87, 1.11]
Ambalavanan 2025	PS ITT with budesonide ITT	99	321	100	318	0.98 [0.78, 1.24]
Total (95% CI)		358	845	369	853	0.98 [0.88, 1.09]
Heterogeneity: Tau² = 0.00; Chi² = 0.00, df = 1 (P = 0.99); I² = 0%<br>Test for overall effect: Z = 0.32 (P = 0.75)						
**Grade 3 (severe)**						
Manley 2024	PS ITT with budesonide ITT	20	524	13	535	1.57 [0.79, 3.12]
Ambalavanan 2025	PS ITT with budesonide ITT	28	321	26	318	1.07 [0.64, 1.78]
Total (95% CI)		48	845	39	853	1.22 [0.81, 1.84]
Heterogeneity: Tau² = 0.00; Chi² = 0.78, df = 1 (P = 0.38); I² = 0%<br>Test for overall effect: Z = 0.97 (P = 0.33)						

Author (year)	Intervention	PS with budesonide		PS (control)		Risk Ratio (95% CI)
		Event	Total	Event	Total	
**Secondary outcomes**						
**Other respiratory outcomes**						
**Postnatal systemic corticosteroid requirement**						
Manley 2024	PS ITT with budesonide ITT	174	524	176	535	1.01 [0.85, 1.20]
Ambalavanan 2025	PS ITT with budesonide ITT	102	321	109	318	0.93 [0.74, 1.16]
Total (95% CI)		276	845	285	853	0.98 [0.85, 1.12]
Heterogeneity: Tau² = 0.00; Chi² = 0.36, df = 1 (P = 0.55); I² = 0%<br>Test for overall effect: Z = 0.33 (P = 0.74)						
**Pulmonary hemorrhage**						
Manley 2024	PS ITT with budesonide ITT	38	524	56	535	0.69 [0.47, 1.03]
Ambalavanan 2025	PS ITT with budesonide ITT	11	322	14	313	0.76 [0.35, 1.66]
Total (95% CI)		49	846	70	848	0.71 [0.50, 1.00]
Heterogeneity: Tau² = 0.00; Chi² = 0.05, df = 1 (P = 0.83); I² = 0%<br>Test for overall effect: Z = 1.94 (P = 0.05)						
**Other preterm outcomes**						
**Pre-discharge mortality**						
Sadeghnia 2018	PS ITT with budesonide NB	4	35	6	35	0.67 [0.21, 2.16]
Manley 2024	PS ITT with budesonide ITT	97	524	114	535	0.87 [0.68, 1.11]
Ambalavanan 2025	PS ITT with budesonide ITT	50	321	44	313	1.11 [0.76, 1.61]
Total (95% CI)		151	880	164	883	0.92 [0.76, 1.13]
Heterogeneity: Tau² = 0.00; Chi² = 1.45, df = 2 (P = 0.48); I² = 0%<br>Test for overall effect: Z = 0.77 (P = 0.44)						
**Late onset sepsis**						
Manley 2024	PS ITT with budesonide ITT	75	524	80	535	0.96 [0.72, 1.28]
Ambalavanan 2025	PS ITT with budesonide ITT	70	322	70	313	0.97 [0.73, 1.30]
Total (95% CI)		145	846	150	848	0.96 [0.78, 1.19]
Heterogeneity: Tau² = 0.00; Chi² = 0.01, df = 1 (P = 0.94); I² = 0%<br>Test for overall effect: Z = 0.34 (P = 0.73)						
**Patent ductus arteriosus requiring treatment**						
Manley 2024	PS ITT with budesonide ITT	138	524	156	535	0.90 [0.74, 1.10]
Ambalavanan 2025	PS ITT with budesonide ITT	85	319	98	308	0.84 [0.66, 1.07]

Author (year)	Intervention	PS with budesonide		PS (control)		Risk Ratio (95% CI)
		Event	Total	Event	Total	
Total (95% CI)		223	843	254	843	0.88 [0.75, 1.02]
Heterogeneity: Tau² = 0.00; Chi² = 0.22, df = 1 (P = 0.64); I² = 0%<br>Test for overall effect: Z = 1.69 (P = 0.09)						
**Adverse effects**						
**Hyperglycemia**						
Manley 2024	PS ITT with budesonide ITT	340	524	331	535	1.05 [0.96, 1.15]
Ambalavanan 2025	PS ITT with budesonide ITT	214	322	156	313	1.33 [1.16, 1.53]
Total (95% CI)		554	846	487	848	1.18 [0.93, 1.49]
Heterogeneity: Tau² = 0.03; Chi² = 8.34, df = 1 (P = 0.004); I² = 88%<br>Test for overall effect: Z = 1.35 (P = 0.18)						
**Spontaneous Intestinal Perforation (SIP)**						
Manley 2024	PS ITT with budesonide ITT	19	524	17	535	1.14 [0.60, 2.17]
Ambalavanan 2025	PS ITT with budesonide ITT	17	322	9	313	1.84 [0.83, 4.06]
Total (95% CI)		36	846	26	848	1.38 [0.84, 2.27]
Heterogeneity: Tau² = 0.00; Chi² = 0.83, df = 1 (P = 0.36); I² = 0%<br>Test for overall effect: Z = 1.26 (P = 0.21)						

Abbreviations: CI: confidence interval; ITT: Intratracheal; NB: nebulization or inhalation; PS: Pulmonary surfactant;

S4 Table. Summary results of the included studies categorized by outcomes (continuous data)

Author (year)	Intervention	PS with budesonide			PS (control)			Mean difference (95% CI)
		Mean	SD	N	Mean	SD	N	
**Duration of mechanical ventilation (days)**								
Manley 2024	PS ITT with budesonide ITT	8.0*	17.78*	524	8.0*	16.3*	535	0.00 [-2.06 to 2.06]
Ambalavanan 2025	PS ITT with budesonide ITT	10.0*	22.96*	315	12.0*	25.19*	304	-2.00 [-5.80, 1.80]
Total (95% CI)				839			839	-0.45 [-2.26, 1.36]
Heterogeneity: Tau² = 0.00; Chi² = 0.82, df = 1 (P = 0.36); I² = 0%<br>Test for overall effect: Z = 0.49 (P = 0.62)								
**Duration of hospitalization (days)**								
Manley 2024	PS ITT with budesonide ITT	110.0*	31.85*	427	110.0*	30.37*	421	0.00 [-4.19 to 4.19]
Ambalavanan 2025	PS ITT with budesonide ITT	96.0*	45.93*	310	95.0*	40.0*	303	1.00 [-5.81, 7.81]
Total (95% CI)				737			724	0.27 [-3.29, 3.84]
Heterogeneity: Tau² = 0.00; Chi² = 0.06, df = 1 (P = 0.81); I² = 0%<br>Test for overall effect: Z = 0.15 (P = 0.88)								

Abbreviations: CI: confidence interval; ITT: Intratracheal; PS: Pulmonary surfactant; SD: standard deviation
* Converted data from median (IQR 1-3) to mean (SD)

S5 Table. List of excluded studies and reasons for exclusion

Author	Year	Study name	DOI	Journal	URL	Reasons for exclusion
Arnon	1996	Effectiveness of budesonide aerosol in ventilator-dependent preterm babies: A preliminary report	10.1002/(sici)1099-0496(199604)21:4<231::Aid-ppul5>3.0.Co;2-r.	Pediatric Pulmonology	https://pubmed.ncbi.nlm.nih.gov/9121852/	- Irrevant<br>- No surfactant was used in the study.
Inwald	1999	The effect of early inhaled budesonide on pulmonary inflammation in infants with respiratory distress syndrome	10.1007/s004310051212	European Journal of Pediatrics	https://link.springer.com/article/10.1007/s004310051212	- Not RCT<br>- This study was not a controlled trial.
Bassler	2010	The Neonatal European Study of Inhaled Steroids (NEUROSIS): an eu-funded international randomised controlled trial in preterm infants	10.1159/000227294	Neonatology	https://pubmed.ncbi.nlm.nih.gov/19590247/	- Protocol<br>- This was a study protocol comparing budesonide with a placebo.
Kuo	2010	A follow-up study of preterm infants given budesonide using surfactant as a vehicle to prevent chronic lung disease in preterm infants.	10.1016/j.jpeds.2009.10.049	The Journal of Pediatrics	https://pubmed.ncbi.nlm.nih.gov/20138301/	- This study was a follow-up study of Yeh 2006.
Wan	2010	Study on combining budesonide suspension, pulmonary surfactant Curosurf and nasal continuous positive airway pressure in treatment of respiratory distress syndrome of premature	-	Pharmaceutical Biotechnology	-	- Irrelevant study<br>- The study included preterm infants, with a mean gestational age of 32–32.5 weeks and a mean birth weight ranging from 1,580 to 1,601 g.
Wu	2012	Budesonide therapy in preterm infants to prevent	10.1542/neo.13-8-e467.	NeoReviews	https://www.scopus.com/inward/record.uri?eid=2-s2.0-84865659819&doi=10.1542%2fne	- Not RCT<br>- This was a review.

Author	Year	Study name	DOI	Journal	URL	Reasons for exclusion
		bronchopulmonary dysplasia			o.13-8-e467&partnerID=40&md5=abc9ab561e0be0ae2c242b162e600b04	
Ke	2016	Efficacy of different preparations of budesonide combined with pulmonary sur-factant in the treatment of neonatal respiratory distress syndrome: A comparative analysis.	10.7499/j.issn.1008-8830.2016.05.005	Chinese Journal of Contemporary Pediatrics	https://pubmed.ncbi.nlm.nih.gov/27165587/	- Irrelevant study<br>- The participants' gestational age was less than 32 weeks, but the mean GA was not reported.
Pan	2017	Clinical efficacy of pulmonary surfactant combined with budesonide for preventing bronchopulmonary dysplasia in very low birth weight infants.	10.7499/j.issn.1008-8830.2017.02.002	Chinese Journal of Contemporary Pediatrics	https://pubmed.ncbi.nlm.nih.gov/28202108/	- Irrelevant study<br>- The study included infants with a gestational age of less than 32 weeks. The participants' mean gestational age was 29.5–30 weeks, and their mean birth weight was 1,260–1,360 g.
Yu	2018	Effects of different forms of budesonide combined with pulmonary surfactant in improving blood gas indicators and bronchopulmonary dysplasia in children with neonatal respiratory distress syndrome.	-	Chinese Journal of Lung Diseases	-	- Irrelevant study<br>- The participants' mean gestational age was 31.4 weeks, and their mean birth weight was 1,830 g.
Du	2019	Budesonide and Poractant Alfa prevent bronchopulmonary dysplasia via triggering SIRT1 signaling pathway.	10.26355/eurrev_201912_19811	European Review for Medical and Pharmacological Sciences	https://pubmed.ncbi.nlm.nih.gov/31858575/	- Irrelevant study<br>- The participants' birth weight criteria were ≤ 1,500 g and gestational age ≤ 32 weeks. However, the mean gestational age and mean birth weight were

Author	Year	Study name	DOI	Journal	URL	Reasons for exclusion
						not available in this study.
McEvoy	2019	Pilot dose escalation trial of budesonide in surfactant in ventilated extremely low gestational age newborns	10.1038/s41390-020-0792-y	Journal of Investigative Medicine	https://pubmed.ncbi.nlm.nih.gov/32006953/	- Irrelevant study<br>- This study compared different doses of budesonide in surfactant for the prevention of bronchopulmonary dysplasia in extremely low gestational age, high-risk newborns. (SASSIE).
Chen	2020	Effect of budesonide on pulmonary surfactant in treating neonatal respiratory distress syndrome.	10.19526/j.cnki.1005-8915.20200314	Pharmaceutical Biotechnology	-	- Irrelevant study<br>- The participants' birth weight criteria were ≤ 1,500 g and gestational age ≤ 32 weeks. However, the mean gestational age and mean birth weight were not provided in this study.
Heo	2020	Intratracheal administration of budesonide with surfactant in very low birth weight infants to prevent bronchopulmonary dysplasia	10.24953/turkjped.2020.04.004	The Turkish Journal of Pediatrics	https://pubmed.ncbi.nlm.nih.gov/32779407/	- Not RCT
McEvoy	2020	Dose-escalation trial of budesonide in surfactant for prevention of bronchopulmonary dysplasia in extremely low gestational age high-risk newborns (SASSIE)	10.1038/s41390-020-0792-y	Pediatric Research	https://www.nature.com/articles/s41390-020-0792-y	- Irrelevant study<br>- This study compared different doses of budesonide, and there was no control group without budesonide.

Author	Year	Study name	DOI	Journal	URL	Reasons for exclusion
Tukova	2020	Early inhaled budesonide in extremely preterm infants decreases long-term respiratory morbidity	10.1002/ppul.24704	Pediatric Pulmonology	https://www.scopus.com/inward/record.uri?eid=2-s2.0-85081016882&doi=10.1002%2fppul.24704&partnerID=40&md5=7fa9dd69b622350b7d7e780a29558cec	- Irrelevant study<br>- This study compared budesonide with a placebo, not budesonide with pulmonary surfactant
Ballard	2021	Blood metabolomics in infants enrolled in a dose escalation pilot trial of budesonide in surfactant	10.1038/s41390-020-01343-z.	Pediatric Research	https://pubmed.ncbi.nlm.nih.gov/33469180/	- Irrelevant study<br>- This study was a pilot dose escalation trial of budesonide in surfactant for intubated premature infants.
Chuanlong	2021	Evaluation of the Efficacy of Budesonide Combined with Pulmonary Surfactants on the Neonatal Respiratory Distress Syndrome by Pulmonary Ultrasonography.	10.1155/2021/2329524	Scientific Programming	https://onlinelibrary.wiley.com/doi/10.1155/2021/2329524	- Irrelevant study<br>- The participants' birth weight criteria were less than 2,500 g and gestational age less than 35 weeks. However, the mean gestational age was 30.1–31.8 weeks, and the mean birth weight was 1,680–1,710 g.
Yao	2021	Efficacy of budesonide in the prevention and treatment of bronchopulmonary dysplasia in premature infants and its effect on pulmonary function.	-	American Journal of Translational Research	https://pubmed.ncbi.nlm.nih.gov/34150079/	- Irrelevant study<br>- The participants' birth weight criteria were < 1,500 g and gestational age 28–$31^{6/7}$ weeks. However, the mean gestational age was 30 weeks, and the mean birth weight was 1,325–1,350 g.
Zheng	2021	Effect of calf pulmonary surfactant for injection combined with budesonide on preven-tion and treatment of neonatal	10.3760/cma.j.cn115689-20210519-01751	Chinese Journal of Practical Internal Medicine	https://rs.yiigle.com/cmaid/1336451	- Irrelevant study<br>- The mean gestational age of the participants was 30.4–31.2 weeks, and the mean birth

Author	Year	Study name	DOI	Journal	URL	Reasons for exclusion
		bronchopulmonary dysplasia.				weight was 1,658–1,662 g.
Liu	2022	Efficacy and safety of intratracheal administration of budesonide combined with pulmonary surfactant in preventing bronchopulmonary dysplasia: A prospective randomized controlled trial.	10.7499/j.issn.1008-8830.2109106	Chinese Journal of Contemporary Pediatrics	https://pubmed.ncbi.nlm.nih.gov/35177180/	- Irrelevant study<br>- The participants' birth weight criteria were < 1,500 g and gestational age < 32 weeks. However, the mean gestational age was 29.3–29.6 weeks, and the mean birth weight was 1,175–1,199 g.
Armanian	2023	Intratracheal Administration of Budesonide-instilled Surfactant for Prevention of Bronchopulmonary Dysplasia: A Randomized Controlled Clinical Trial.	10.22038/IJN.2023.72572.2406	Iranian Journal of Neonatology	https://ijn.mums.ac.ir/article_22919_b4ad1dcf2df8d574fefd90bedad92cd8.pdf	- Irrelevant study<br>- The participants' gestational age criteria were < 30 weeks. However, the mean gestational age was 28.9–29 weeks, and the mean birth weight was 1,134–1,190 g.
Gurung	2023	Neurodevelopmental outcomes in extremely premature infants treated with intratracheal budesonide-surfactant for the prevention of bronchopulmonary dysplasia	10.1016/S0002-9629(23)00609-2	American journal of the medical sciences	https://www.cochranelibrary.com/central/doi/10.1002/central/CN-02522137/full	- Not RCT<br>- This was a retrospective study.
Kashaki	2023	Simultaneous intratracheal administration of surfactant and budesonide in the prevention of bronchopulmonary dysplasia	10.62940/als.v10i2.1856	Advancements in Life Sciences |	https://www.als-journal.com/articles/vol10issue2/10225.23/1856.pdf	- Irrelevant study<br>- The participants’ mean gestational age was 29.6–30.2 weeks.

Author	Year	Study name	DOI	Journal	URL	Reasons for exclusion
		in the treatment of respiratory distress syndrome in premature infants				
Manley	2023	Intratracheal budesonide mixed with surfactant to increase survival free of bronchopulmonary dysplasia in extremely preterm infants: study protocol for the international, multicenter, randomized PLUSS trial	10.1186/s13063-023-07257-5	Trials, 2023, 24(1), 320 | added to CENTRAL: 31 May 2023 | 2023 Issue 5	https://www.cochranelibrary.com/central/doi/10.1002/central/CN-02562155/full	- Protocol<br>- This was a study protocol comparing budesonide with surfactant to surfactant alone.
Safa	2023	Budesonide and surfactant combination for treatment of respiratory distress syndrome in preterm neonates and evaluation outcomes.	10.4103/jcn.jcn_52_23	Journal of Clinical Neonatology	https://journals.lww.com/jocn/fulltext/2023/12040/budesonide_and_surfactant_combination_for.1.aspx	- Irrelevant study<br>- The participants' gestational age criteria were <37 weeks and birth weight between 800–1,500 g. However, the mean gestational age was 29.3–29.9 weeks, and the mean birth weight was 1,139–1,186 g.
Dini	2024	Strategies for the prevention of bronchopulmonary dysplasia(Short Survey)	10.3389/fped.2024.1439265	Frontiers in Pediatrics	https://www.scopus.com/inward/record.uri?eid=2-s2.0-85200680776&doi=10.3389%2ffped.2024.1439265&partnerID=40&md5=bf422b4cbcd2b21e0db9e72d1b14e309	- Not RCT,<br>- This was a review article.
Habibi	2024	Comparison of the Consequences of Intratracheal Administration of Surfactant Plus Budesonide with Surfactant alone in Preterm Infants with	10.61186/sjku.28.6.48	Scientific Journal of Kurdistan University of Medical Sciences	http://sjku.muk.ac.ir/article-1-7342-en.html	- Irrelevant study<br>- The mean gestational age of the participants was approximately 30 weeks.

Author	Year	Study name	DOI	Journal	URL	Reasons for exclusion
		Respiratory Distress Syndrome				
IRCT20240524061883N1	2024	The effect of intratracheal surfactant with budesonide in the treatment of respiratory distress syndrome	-	Iranian Registry of Clinical Trials	https://irct.behdasht.gov.ir/trial/77035	- This is an ongoing study that includes babies with a gestational age of 28–34 weeks.
Jensen	2024	Intratracheal Budesonide Combined With Surfactant in Extremely Preterm Infants	10.1001/jama.2024.19641	JAMA	https://pubmed.ncbi.nlm.nih.gov/39527043/	- Not RCT<br>- This was an editorial.
Kugelman	2024	Striving for healthy lungs: Enhancing respiratory outcomes of prematurity	10.1002/ppul.27309	Pediatr Pulmonol.	https://jamanetwork.com/journals/jama/article-abstract/2826120#google_vignette	- Not RCT<br>- This was a review article.
Marzban	2024	The impact of combined admin- istration of surfactant and intratracheal budesonide compared to surfactant alone on bronchopulmonary dysplasia (BPD) and mortality rate in preterm infants with respiratory distress syndrome: a single-blind randomized clinical trial.	10.1186/s12887-024-04736-9	BMC Pediatr	https://bmcpediatr.biomedcentral.com/articles/10.1186/s12887-024-04736-9	- Irrelevant study<br>- The participants' gestational age criteria were <37 weeks and birth weight less than 2,500 g.<br>The mean gestational age of the participants was 31–31.6 weeks, and the mean birth weight was 1,465–1,584 g.
Nobile	2024	Long-term Effects of Intratracheal Budesonide and Surfactant for the Prevention of Bronchopulmonary Dysplasia: A Narrative Review.	10.1055/s-0043-1769795	American journal of perinatology	https://pubmed.ncbi.nlm.nih.gov/37279790/	- Not RCT<br>- This was a systematic review study.
Pereira-Fantini	2024	A complex inflammatory mix: chorioamnionitis,	10.1038/s41390-024-03219-y	Pediatric Research	https://findanexpert.unimelb.edu.au/scholarlywork/1888124-a-	- Not RCT

Author	Year	Study name	DOI	Journal	URL	Reasons for exclusion
		antenatal steroids and early postnatal budesonide			complex-inflammatory-mix--chorioamnionitis--antenatal-steroids-and-early-postnatal-budesonide	
Prodanovic	2024	Advanced Diagnostics of Respiratory Distress Syndrome in Premature Infants Treated with Surfactant and Budesonide through Computer-Assisted Chest X-ray Analysis	10.3390/diagnostics14020214	Diagnostics	https://www.mdpi.com/2075-4418/14/2/214	- Not RCT<br>- This study was not an RCT; instead, it compared outcomes before and after receiving surfactant.
Xu	2024	Therapeutic Efficacy of Budesonide Suspension Combined with Poractant Alfa Injection for Neonatal Respiratory Distress Syndrome and its Effect on Serum Ferritin and PAI-1 Expressions	-	Latin American Journal of Pharmacy	https://www.scopus.com/inward/record.uri?eid=2-s2.0-85191477077&partnerID=40&md5=9a25cdf9d336d8aa1daee5f94cf30d02	- Not RCT and irrelevant,<br>This study was described as an RCT but was only divided into an observed and a control group. Additionally, the results were not relevant to our objectives and outcomes.
Adow	2025	EBNEO Commentary: Intratracheal Budesonide Mixed With Surfactant for Extremely Preterm Infants. The PLUSS Randomized Clini cal Trial	10.1111/apa.70298	Acta Paediatrica, International Journal of Paediatrics - Volume 0, Issue 0, pp. - published 2025-01-01	https://www.scopus.com/inward/record.uri?eid=2-s2.0-105016614187&doi=10.1111%2Fapa.70298&partnerID=40&md5=df0fd499f420ce1b1866b9529f9469ad	- Not RCT, commentary
Ahmad	2025	Effectiveness of Intratracheal Surfactant Mixed With Budesonide for Prevention of Bronchopulmonary Dysplasia	10.1002/ppul.71114	Pediatric Pulmonology	https://www.scopus.com/inward/record.uri?eid=2-s2.0-105006413788&doi=10.1002%2Fppul.71114&partnerID=40&md5=00ced7397fecf12c299def6d82cf2bbe	- Not RCT, letter to editor

Author	Year	Study name	DOI	Journal	URL	Reasons for exclusion
Jin	2025	Less invasive surfactant administration combined with budesonide in preterm infants with respiratory distress syndrome : a clinical research	10.3760/cma.j.issn.1671-0282.2025.06.008	Chinese Journal of Emergency Medicine	https://www.scopus.com/inward/record.uri?eid=2-s2.0-105011370021&doi=10.3760%2Fcma.j.issn.1671-0282.2025.06.008&partnerID=40&md5=1a5ab4c1bd9718f737df6aeaa68ac3f3	- Irrelevant, age cover more than 28 weeks
Lima	2025	Does intratracheal budesonide mixed with surfactant increase survival without bronchopulmonary dysplasia in extremely preterm infants?	10.1038/s41372-025-02391-1	Journal of Perinatology	https://www.scopus.com/inward/record.uri?eid=2-s2.0-105013462242&doi=10.1038%2Fs41372-025-02391-1&partnerID=40&md5=1adb1cdfccf276ab9479282683780993	- Not RCT
Pillai	2025	Intratracheal Administration of Budesonide with Surfactant for Prevention of Death or Bronchopulmonary Dysplasia in Extremely Low Birth Weight Infants: A before-after Study	10.1055/a-2506-2893	American Journal of Perinatology	https://www.scopus.com/inward/record.uri?eid=2-s2.0-85216778711&doi=10.1055%2Fa-2506-2893&partnerID=40&md5=15e2d59b5322fce02837dbee9e9ae265	- Not RCT
Russian	2025	Surfactant Combined With Budesonide: A Review of the Evidence	-	Neonatal Intensive Care	https://www.embase.com/search/results?subaction=viewrecord&id=L2038287638&from=export U2 - L2038287638	- Not RCT, review
Seifert	2025	Intratracheal budesonide in addition to surfactant. The PLUSS for extremely preterm infants?	-	Krankenhauspharmazie	https://www.scopus.com/inward/record.uri?eid=2-s2.0-105005876939&partnerID=40&md5=3aaa99f3417ae1a01b64b2539637144d	- Not RCT
Zhang	2025	An excerpt of Intratracheal Budesonide Mixed with Surfactant for Extremely Preterm Infants: the	10.3760/cma.j.cn101451-20241216-00426	Chinese Journal of Neonatology	https://www.scopus.com/inward/record.uri?eid=2-s2.0-105005755425&doi=10.3760%2Fcma.j.cn101451-20241216-00426&partnerID=40&md5=c63d7632f83e4b73c9d65c59b1c333ee	- Not RCT, an excerpt

Author	Year	Study name	DOI	Journal	URL	Reasons for exclusion
		PLUSS Randomized Clinical Trial				

Abbreviations: RCT: randomized controlled trial
