# Supplementary material for: Efficacy of pulmonary surfactant with budesonide in infants born at or less than 28 weeks’ gestation: a systematic review and meta-analysis
Source: Sci Rep. 2025 Dec 22;15:45116. doi: 10.1038/s41598-025-33028-0 (PMC12748727; doi:10.1038/s41598-025-33028-0)

S1 Fig. Risk of bias summary of the included studies using the revised Cochrane risk of bias tool for randomized trials assessing incidence of bronchopulmonary dysplasia using National Institutes of Health (NIH) consensus definition or Jensen et al. 2019 definition

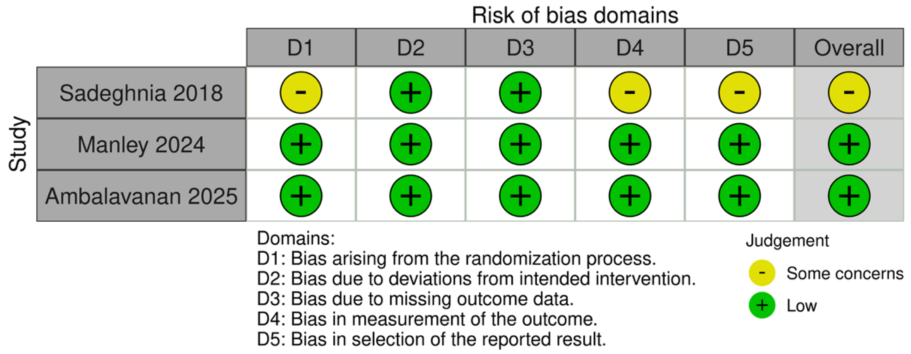

S2 Fig. Risk of bias summary of the included studies using the revised Cochrane risk of bias tool for randomized trials assessing severity of bronchopulmonary dysplasia according to Jensen et al. 2019 definition

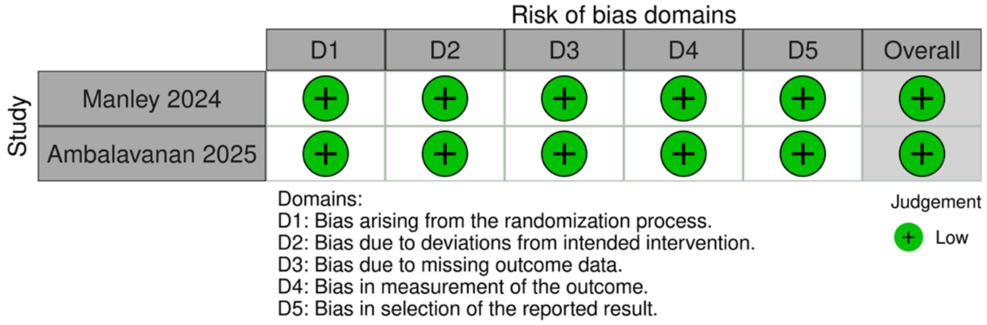

S3 Fig. Risk of bias summary of the included studies using the revised Cochrane risk of bias tool for randomized trials assessing postnatal systemic corticosteroid requirement

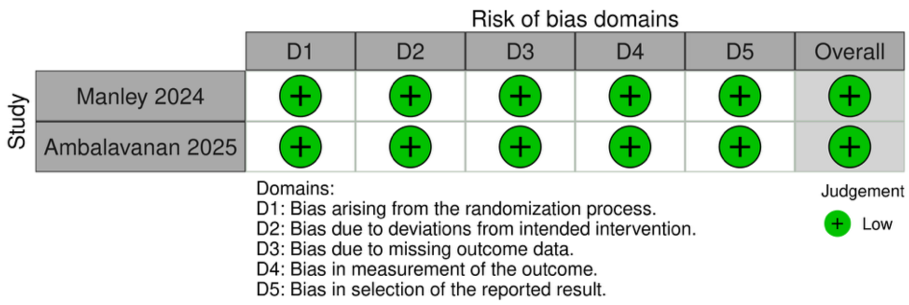

S4 Fig. Risk of bias summary of the included studies using the revised Cochrane risk of bias tool for randomized trials assessing pulmonary hemorrhage

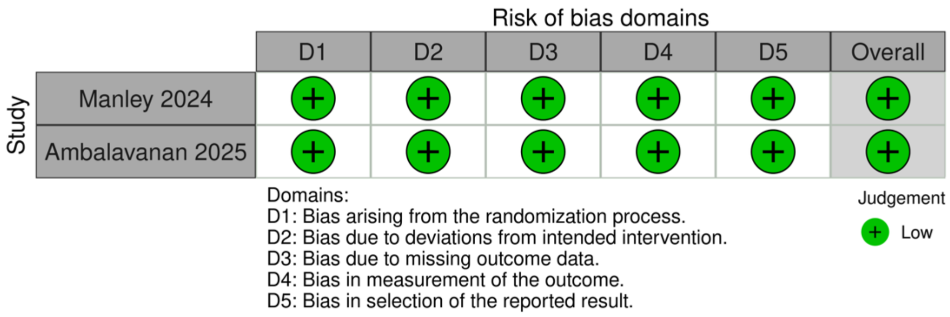

S5 Fig. Risk of bias summary of the included studies using the revised Cochrane risk of bias tool for randomized trials assessing pre-discharge mortality

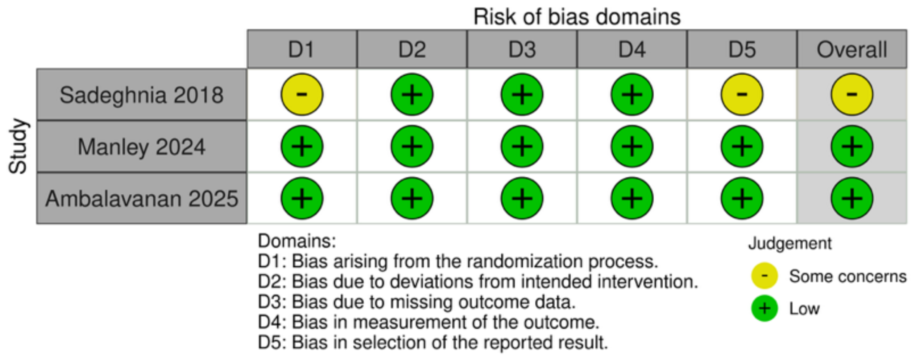

S6 Fig. Risk of bias summary of the included studies using the revised Cochrane risk of bias tool for randomized trials assessing late onset sepsis

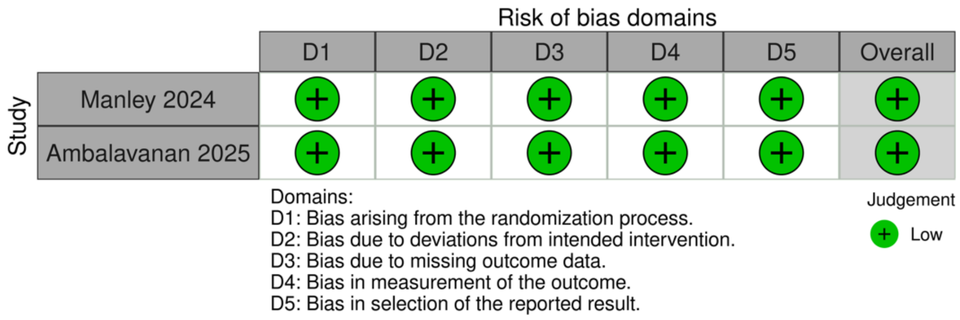

S7 Fig. Risk of bias summary of the included studies using the revised Cochrane risk of bias tool for randomized trials assessing patent ductus arteriosus requiring treatment

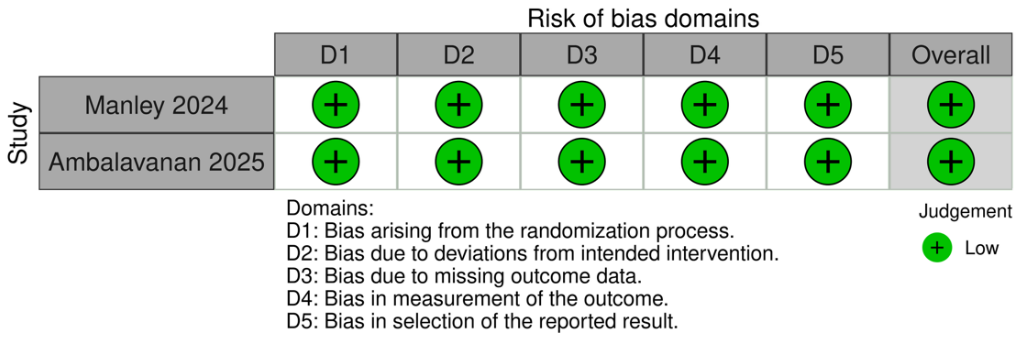

S8 Fig. Risk of bias summary of the included studies using the revised Cochrane risk of bias tool for randomized trials assessing the duration of mechanical ventilation (days)

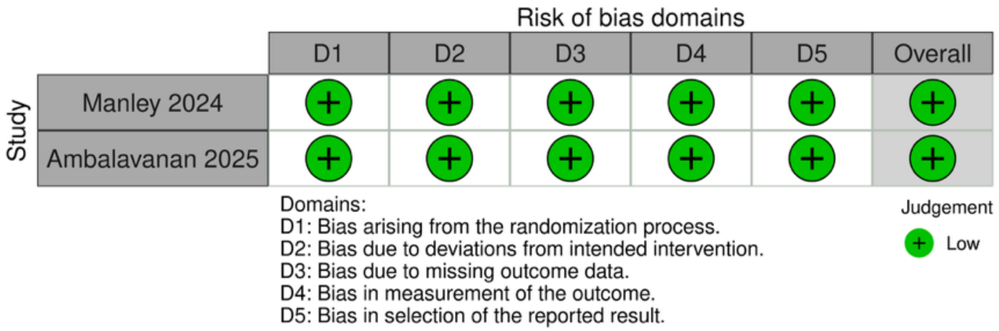

S9 Fig. Risk of bias summary of the included studies using the revised Cochrane risk of bias tool for randomized trials assessing the duration of hospitalization (days)

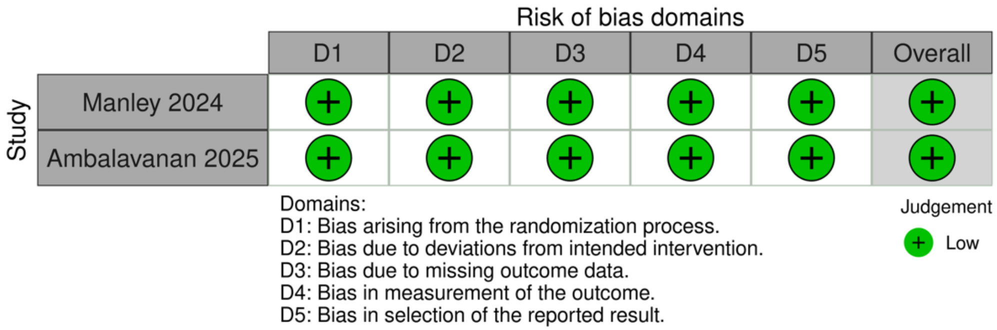

S10 Fig. Risk of bias summary of the included studies using the revised Cochrane risk of bias tool for randomized trials assessing hyperglycemia

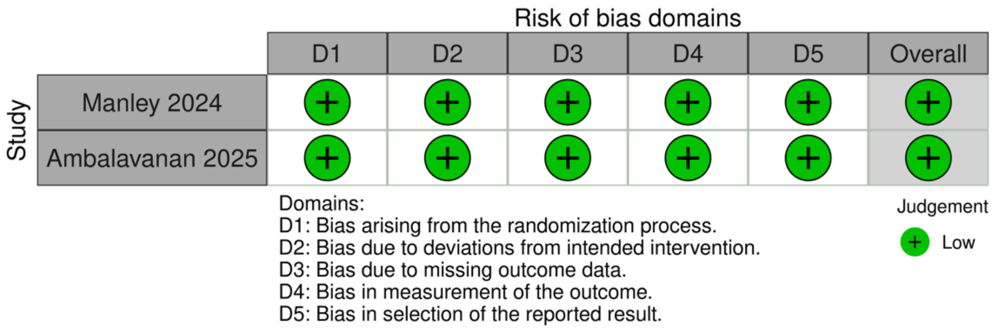

S11 Fig. Risk of bias summary of the included studies using the revised Cochrane risk of bias tool for randomized trials assessing spontaneous intestinal perforation (SIP)

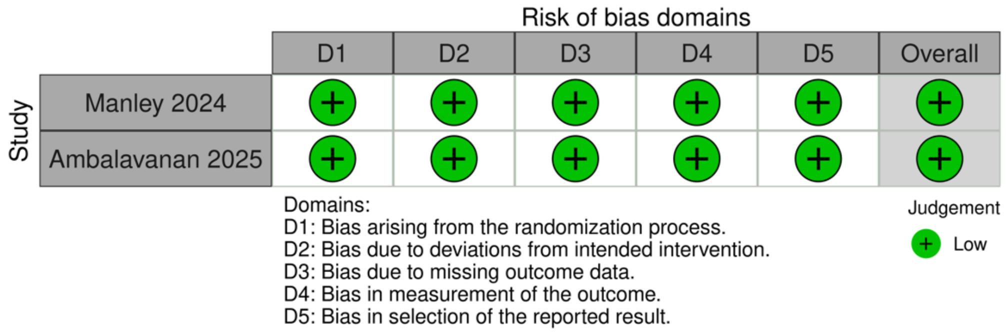

Supplement: Supplementary file 2 — Supplementary Material 2 [file 41598_2025_33028_MOESM2_ESM.pdf]
